# Collagenous Gastritis in Primary Selective IgM Deficiency: Transition to EBV+ Gastric Adenocarcinoma

**DOI:** 10.1155/2021/5574944

**Published:** 2021-05-25

**Authors:** Tejal Narsai, Houfen Su, David Braxton, Sudhir Gupta

**Affiliations:** ^1^Division of Basic and Clinical Immunology, University of California, Irvine, California, USA; ^2^Hoag Hospital, Newport Beach, California, USA

## Abstract

Selective IgM deficiency (SIgMD) and isolated collagenous gastritis are two independent rare disorders. Our purpose is to report the 1^st^ case of SIgMD and isolated collagenous gastritis and collagenous gastritis that has transitioned to EBV + gastric adenocarcinoma. Gastric biopsy tissue was analyzed by EBV-related encoded RNA in situ hybridization assay. Subsets of CD4, CD8, T follicular helper cells (T_FH_), and members of the “regulatory lymphocytes club” were measured with multiple panels of monoclonal antibodies and isotype controls by multicolor flow cytometry. The patient was diagnosed with SIgMD (extremely low serum IgM 9 mg/dl and normal IgG and IgA and exclusion of secondary causes of low IgM). Soon after SIgMD diagnosis, the patient developed collagenous gastritis and, 8 years later, developed gastric adenocarcinoma that was positive for EBV. An extensive immunological analysis revealed reduced naïve CD4 and CD8 effector memory T cells and increased naïve and central memory CD8 T cells. Among the circulating follicular helper T cells (cT_FH_), T_FH_1 and T_FH_2 were increased whereas T_FH_17 was decreased. CD4 Treg cells and T_FR_ cells were increased, whereas Breg and CD8 Treg were comparable to control. In conclusion, SIgMD may be associated with isolated collagenous gastritis, and collagenous gastritis may transition to EBV + gastric adenocarcinoma. A role of regulatory lymphocytes in gastric cancer is discussed.

## 1. Introduction

SIgMD was first described in 1967 [1]; however, only recently has it been incorporated as a primary immunodeficiency in IUIS classification [2]. SIgMD is characterized by a serum IgM below 2 SD below the mean with normal serum IgG and IgA, and exclusion of secondary causes of low serum IgM [3]. Patients with SIgMD may be asymptomatic or present with recurrent infections and allergic and/or autoimmune manifestations [4, 5]. A number of malignant disorders have been reported in patients with SIgMD [6, 7]; however, it is unclear whether there is a true increase in the prevalence of malignancy in SIgMD.

Collagenous gastroenteritis includes collagenous gastritis, collagenous sprue, and collagenous colitis and is characterized by subepithelial collagen deposition and infiltration by inflammatory mononuclear cells in the lamina propria [8–12]. Among collagenous gastroenteritides, collagenous gastritis is very rare and isolated collagenous gastritis is predominantly present in children. In adults, it is generally associated with diffused disease including collagenous colitis [13]. Kamimura et al. reviewed data on all 60 known cases of collagenous gastritis reported until 2015, and no progression of collagenous gastritis to gastric carcinoma was observed [14].

Collagenous gastritis has not been reported as a predisposing factor for gastric cancer, and collagenous gastritis has not been reported in SIgMD. Furthermore, progression of collagenous gastritis to gastric adenocarcinoma has never been reported.

We present, to the best of our knowledge, the first case of SIgMD with isolated collagenous gastritis and transition of collagenous gastritis to gastric adenocarcinoma.

## 2. Materials and Methods

### 2.1. Case Description

**Figure 1:**
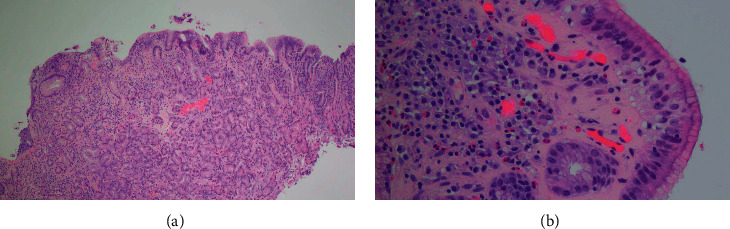
(a) 4x magnification of the results of upper endoscopy biopsy showing nonspecific chronic inflammatory infiltrate with a thickened subepithelial collagen table. (b) 20x magnification higher-power image of the thickened subepithelial collagen table, showing diagnostic features of entrapped cellular elements such as inflammatory cells and blood vessels.

**Figure 2:**
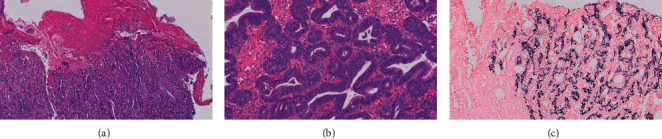
(a) 10x magnification of histopathology of the polypoid gastric body lesion showing poorly differentiated adenocarcinoma with surface ulceration. (b) High-power magnification of the gastric polypoid lesion showing high-grade dysplasia. (c) EBV-infected carcinoma cells (black stain present) juxtaposed with normal gastric glandular mucosa (black stain absent), using EBV-related encoded RNA in situ hybridization assay (EBER ish).

In 2017, a 53-year-old male was referred to us with a history of asthma and allergic rhinitis and history of recurrent upper respiratory tract infections. During his teenage years, he reported having frequent episodes of acute sinusitis. In his 30 s, he was diagnosed with an episode of meningitis, as well as multiple pneumonias. No further details were available regarding nature of infections. No prior immunological workup was performed. His asthma was well controlled on inhaled corticosteroid therapy. His allergic rhinitis was well controlled with allergen immunotherapy and nasal fluticasone spray. An immunologic evaluation was performed. the patient had severely reduced IgM (9 mg/dl; control 37–336) with normal IgG (698 mg/dl; control 660–1,660 mg/dl) and IgA (145 mg/dl; control 80–400 mg/dl) and normal response to pneumococcal polysaccharide, diphtheria, and tetanus toxoid. CD3+, CD4+, CD8+, CD19+ B cells, and CD3-CD16+CD56+ NK cells were normal; secondary causes of low IgM were excluded. Therefore, a diagnosis of primary SIgMD was established. Since 2017, immunoglobulin levels were frequently repeated on several occasions. Total IgG ranged between 698 mg/dl–718 mg/dl, and total IgM ranged between <9 mg/dl and 17 mg/dl. Soon after, he was evaluated for epigastric burning and severe upper abdominal pain. An endoscopy was performed, and biopsies showed collagenous gastritis (Figure 1). He was started on twice daily proton pump inhibitor and H2 blocker therapy, with relief of his symptoms. His gastritis was monitored with annual endoscopies with random biopsies. Eight years later (2020), his screening endoscopy showed a new polyp that, on biopsy, revealed poorly differentiated gastric adenocarcinoma that was positive for EBV (Figure 2). Serum EBV-VCA IgM antibodies (U/ml)- undetected, EBV-VCA IgG antibodies (U/ml)-388 (control <22), EBNA-IgG antibodies (U/ml)- 55.2 (control <22), EBV-EA diffuse antibodies (U/ml)-<5 (control <11), and EBV-PCR-negative were found. He had no new symptoms. A PET CT was negative for metastatic lesion. He underwent total gastrectomy and had 0/22 positive lymph nodes. No chemotherapy was instituted. He is doing well clinically.

### 2.2. Sample Preparation

Peripheral blood was drawn from the patient following the diagnosis of gastric adenocarcinoma and from age- and gender-matched control. Peripheral blood mononuclear cells (PBMCs) were isolated from blood by using density gradient lymphocyte separation media. Human Subject Committee of the Institution Review Board of the University of California, Irvine, approved the protocol. Signed written consent was obtained.

### 2.3. Antibodies and Reagents

The following anti-human monoclonal antibodies and isotype controls were purchased from BD Biosciences (San Jose, California): CD4 PerCP, CD8 PerCP, CD45RA APC, CCR7 FITC, CD183 PE, CD25 FITC, CD127 AL647, FoxP3 PE, CD278 (ICOS) AL647, CD183 BV421, CXCR5 AL488, PD1 APC, CD8 BV421, CD45RA BV510, CD19 PerCP, CD38 FITC, and CD24 FITC.

### 2.4. Flow Cytometry

Approximately 1 million PBMCs were used per combination for antibody staining. 20 *μ*l of antibody was added to PBMCs for 30 min. PBMCs were washed and fixed by 2% paraformaldehyde (PFA).

For regulatory cells, the following surface staining cells were fixed and permeablized by using a Foxp3 staining buffer set (BD Bioscience, San Jose, California) as per the manufacturer's protocol. Intracellular staining with anti-Foxp3PE monoclonal antibody, and appropriate isotype control (Mouse IgG1k-PE), was used for nonspecific staining.

All flourescence minus one controls and isotype controls were stained and fixed by 2% PFA for flow cytometry. Cells were acquired by using the BD FACS Celesta (Becton-Dickenson, San Jose, CA) equipped with a BVR laser. Forward and side scatters and singlets were used to gate and exclude cellular debris. Thirty thousand cells were acquired and analyzed using FLOWJO software (Ashland, OR).

The following surface makers identified various lymphocyte subsets:

  Subsets of CD4 T cells and CD8+ T cells: naïve (TN)-CD4+/CD8+CD45RA+CCR7+, central memory (TCM)-CD4+/CD8+CD45RA-CCR7+, effector memory (TEM)-CD4+/CD8+CD45RA-CCR7-, CD45RA+effector memory, and terminally differentiated effector memory (TEMRA)-CD4+/CD8+CD45RA+CCR7-
  Subsets of T follicular helper cells: cTFH-CD4+CXCR5+CD45RA-, TFH1-CD4+CXCR5+CD45RA-CCR6-CXCR3+, TFH2-CD4+CXCR5+CD45RA-CCR6-CXCR3, TFH17-CD4+CXCR5+CD45RA-CCR6+CXCR3, and TFH1+TFH17-CD4+CXCR5+CD45RA-CCR6+CXCR3+
  Regulatory lymphocytes: CD4Treg–CD4+CD25+CD127- Foxp3+; CD8 Treg-CD8+CD183+CCR7+CD45RA-FoxP3+; TFR-CD4+CCR5+CD45RA-CD25highFoxP3+; and Breg-CD19+CD24+CD38+

## 3. Results

### 3.1. Subsets of CD4 and CD8 T Cells

**Figure 3:**
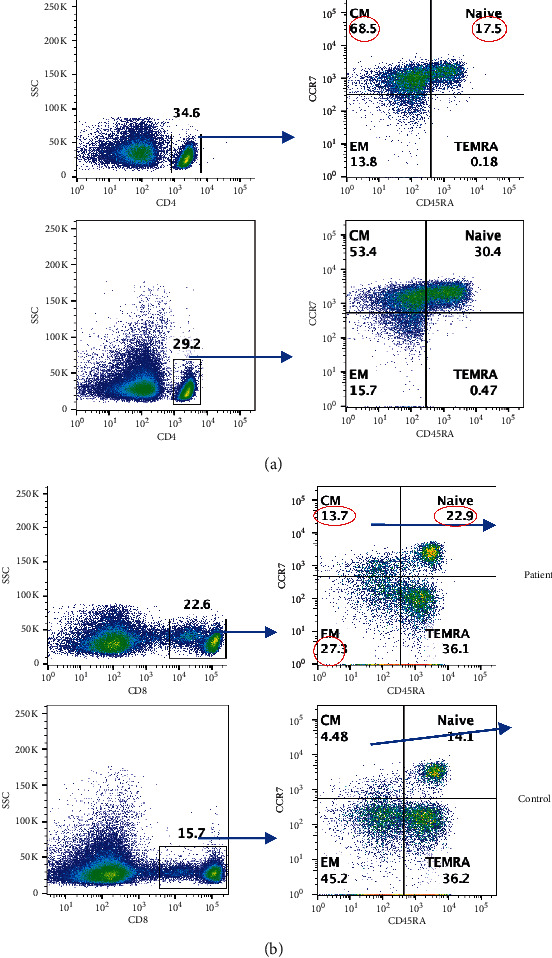
(a) CD4 subsets: CD4+ subsets are characterized by different makers; naïve (TN; CCR7+CD45RA+) central memory: TCM (CCR7+CD45RA-), effector memory: TEM (CCR7-CD45RA-), T effector memory RA: TEMRA (CCR7-CD45RA+). (b) CD8 subset: CD8+ gated cells. In PBMCs, CD88+ T cells were gated and gated CD8+ cells subsets are characterized by different makers: TN (CCR7+ CD45RA+), TCM (CCR7+CD45RA-), TEM (CCR7-CD45RA-), and TEMRA (CCR7-CD45RA+). Abnormal values are circled in red.

Naive T cells (T_N_) upon activation with an antigen undergo clonal expansion and differentiation to effector cells, and at the end of immune response, they are retained as memory T cells. Based on their homing properties, expression of adhesion molecules, and chemokine receptors, memory T cells are classified into central memory (T_CM_) and effector memory (T_EM_) CD4+ and CD8+ T cells [15, 16]. A small population of T_EM_ cells reacquire CD45RA and are termed as terminally differentiated effector memory T cells (T_EMRA_). These subsets differ with regard to proliferative response, cytokine production, effector properties, and sensitivity to apoptosis [15]. Therefore, we examined these subsets in our patient. CD4 T_N_ were decreased, and CD4 T_CM_ were increased (Figure 3(a)). CD8 T_N_ and T_CM_ increased, whereas T_EM_ was decreased (Figure 3(b)).

### 3.2. Subsets of Follicular Helper T Cells

**Figure 4:**
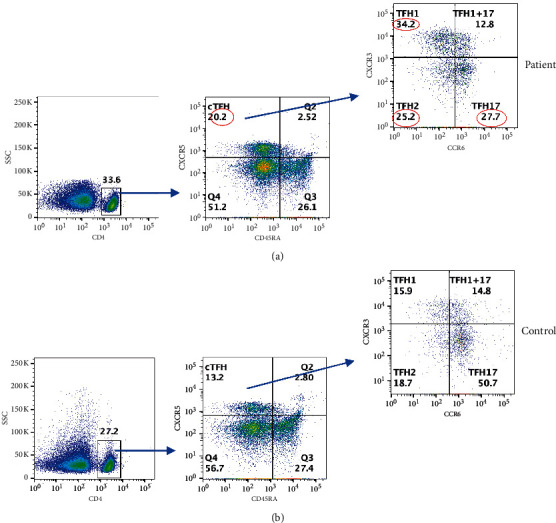
TFH cells: in PBMCs, CD4+ gated cells and various TFH subsets were characterized by different makers: cTFH-CXCR5+CD45RA- and TFH subsets TFH1 (CXCR3+CCR6-), THF1+THF17 (CXCR3+CCR6+), TFH2 (CXCR3-CCR6), and TFH17 (CXCR3-CCR6+). Abnormal values are circled in red. (a) CD4+T-cell subsets. (b) CD8+T-cell subsets.

Circulating T_FH_ cells (cT_FH_) play an important role in germinal center formation, immunoglobulin isotype switching, and differentiation of B cells to immunoglobulin-secreting cells [17, 18]. The signature cytokine they produce is IL-21. However, based on additional cytokines produced, cT_FH_ has been further classified into T_FH_1, T_FH_2, and T_FH_17 [19]. Therefore, we examined all subsets of cT_FH_. cT_FH_, T_FH_1, and T_FH_2, whereas T_FH_17 was reduced as compared to control (Figure 4).

### 3.3. Regulatory Lymphocytes

**Figure 5:**
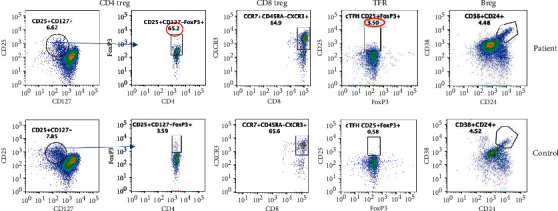
Regulatory lymphocytes: CD4Treg gated CD4+ cells for CD25+CD127- and then analyzed as CD4+CD25+CD127-Foxp3+ cells. Abnormal values are circled in red. CD8 Treg: gated CCR7+CD25highCD45RA- CD8 T cells expressing CD183 (CXCR3) and FoxP3. TFR cells were characterized as TFR-CD4+CCR5+CD45RA-CD25highFoxP3+ and Breg as CD19+CD24+CD38+.

There are 4 members of the “regulatory club” [20–24]. CD4 Treg plays an important role in immune tolerance and cancer [22]. In addition, T follicular regulatory cells (T_FR_) regulate the function of cT_FH_ cells [20, 21]. In addition, CD8 Treg and Breg have also been shown to play a role in peripheral tolerance in cancer [23, 24]. Therefore, we examined all 4 regulatory lymphocytes. T_FR_ cells and CD4 Treg were increased, whereas B reg and CD8 Treg were comparable to control (Figure 5).

## 4. Discussion

SIgMD is a rare primary immunodeficiency disease characterized by low serum IgM and normal IgG and IgA; B cells with surface membrane IgM are normal [6]. We present the 1^st^ SIgMD patient who developed collagenous gastritis that transitioned to EBV + gastric adenocarcinoma.

Collagenous infiltrative disorders of the gastrointestinal tract are characterized by subepithelial deposition of collagen bands with mononuclear cell infiltration in the mucosa [25]. In 1989, Colleti and Trainer [26] reported the first case of collageneous gastritis in a 15-year-old girl who presented with recurrent abdominal pain and bleeding. Collagenous gastritis is extremely rare; since 1989, less than 70 cases of collagenous gastritis have been reported. A few cases of collagenous gastroenteritis have been reported in primary immunodeficiency diseases [27–31]; however, isolated collagenous gastritis has been reported only in one case of hypogammaglobulinemia [32] and in one case of selective IgA deficiency [33]. Ours is the first case of collagenous gastritis in SIgMD. The pathogenesis of collagenous disorders of the gastrointestinal tract remains unclear. A role of the immune system has been proposed based on collagenous gastroenteritis in autoimmune diseases including systemic lupus erythematosus, Sjogren's syndrome, celiac disease, and ulcerative colitis [34–39]. Freeman reported celiac disease in more than 20% of patients with collagenous colitis, a rate that exceeds the reported detection rates of celiac disease in other clinical settings [40].

In a long-term follow-up of patients with collagenous gastritis ranging from 2–16 years, no case of gastric cancer has been observed [41, 42]. However, colon cancer has been rarely recorded in collagenous colitis [43]. Also intriguing was the coincidental later development of lymphomas in 2 patients with collagenous colitis in the absence of celiac disease [40]. Previous reports have recorded Hodgkin and non-Hodgkin lymphomas, including a mycosis fungoides-type T-cell lymphoma in collagenous colitis [44–46]. Additional studies will be needed to determine if there is an increased risk for these lymphoproliferative malignancies in collagenous colitis.

Gastric cancer is the fourth most common cancer and the second leading cause of death worldwide [47]. Gastric cancer is the most common cause of death among CVID patients [48]. Epstein–Barr virus (EBV) is detected in 10% of gastric adenocarcinoma patients [49–54]. Hepatitis B virus (HBV) and *Helicobacter pylori* (*H. Pylori*) have also been implicated in gastric cancer [49]. Kamimura et al. [42] reviewed all 60 patients of collagenous gastritis reported in the world literature until 2015 with a follow-up ranging from 2–14 years. They reported 6 adults and 4 children with collagenous gastritis that were positive for *H. pylori.* None of the patients with collagenous gastritis have ever progressed to gastric cancer. Our patient was negative for *H.* pylori infection. In addition to *H. pylori* and EBV, other predisposing factors for gastric cancer include atropic gastritis and pernicious anemia. Gastric malignancy has not been described in SIgMD. Gastric adenocarcinoma has been reported in patients with other primary immunodeficiencies; however, none were reported to be EBV+ [48, 55–62]. Our patient was diagnosed with collagenous gastritis eight years prior to the development of EBV + adenocarcinoma. Furthermore, at the time of diagnosis, no EBV viremia was present.

In order to understand a role of immune responses in gastric cancer in our patient, we examined various subsets of CD4+ and CD8+ T cells and regulatory lymphocytes. Zhang et al [63] reported increased TFH1 cells that promote inflammation, suppress Breg, and correlate with worse clinical outcome in gastric cancer. Our patient, who had mild course of the disease, also had increased TFH1 cells, but normal Breg cells, suggesting Breg may play a role in clinical outcome of gastric adenocarcinoma. Murakami et al. [64] reported increased regulatory B cells in gastric cancer and suggested that Breg may play a role in immune evasion in gastric cancer. In contrast, Hu et al. [65] reported that IL-10-expressing B cells (Breg) were highly enriched in tumor-infiltrating B cells and were present at reduced frequencies in circulating B cells. Furthermore, they demonstrated that these Breg suppressed cytokine production by CD4+ (IFN-*γ*, TNF*α*, and IL-17) and CD8+ T cells (IFN-*γ* and TNF-*α*). Wang et al. [66] also observed that Breg suppressed TH1 CD4+ T cells (IFN-*γ*) and induced CD4+ Treg and suggested that increased CD4 Treg might contribute to immune escape in gastric cancer. However, in our patient, CD4 Treg was increased and Breg was comparable to control, yet he had a favorable outcome. In SIgMD, Breg and CD8 Treg are increased, whereas CD4 Treg is comparable to control [67]. Therefore, changes in regulatory lymphocytes in our patient are distinct from those in SIgMD and may suggest their role in transition of collagenous gastritis to gastric adenocarcinoma.

## 5. Conclusions

In summary, we described the first case of SIgMD with isolated collagenous gastritis that transitioned to gastric adenocarcinoma. Furthermore, this is the 1st case of EBV + gastric adenocarcinoma in any primary immunodeficiency. The role of immunological alterations in transition of collagenous gastric to EBV + gastric adenocarcinoma is unclear; however, regulatory lymphocytes may play a role in clinical outcome.

## Data Availability

Readers can access the data supporting the conclusions of this study by requesting from the corresponding author.
